# Angiotensin-Converting Enzyme Inhibitors, Angiotensin II Receptor Blockers, and the Risk of Acute Pancreatitis: A Systematic Review and Meta-Analysis

**DOI:** 10.7759/cureus.93136

**Published:** 2025-09-24

**Authors:** Carlos Alves, Beatriz Costa, Ana Penedones, Diogo Mendes, Francisco Batel Marques

**Affiliations:** 1 Laboratory of Social Pharmacy and Public Health, Faculty of Pharmacy, University of Coimbra, Coimbra, PRT; 2 Evidence Review, Synthesis, and Generation, Clevidence, Lda., Oeiras, PRT

**Keywords:** acute pancreatitis, angiotensin-converting enzyme inhibitors, angiotensin ii receptor blockers, meta-analysis, safety, systematic review

## Abstract

Observational studies have evaluated the risk of acute pancreatitis associated with drugs acting on the Renin-Angiotensin-Aldosterone System (RAAS) but reported conflicting results. This meta-analysis aims to investigate the risk of acute pancreatitis associated with angiotensin-converting enzyme (ACE) inhibitors and angiotensin II receptor blockers (ARBs). PubMed and Embase were searched for observational studies. A random-effects model was used to pool odds ratios (ORs). A sensitivity analysis explored the robustness of the initial findings according to study design, treatment duration, dose, age, active substance, and Risk of Bias scores. Main results were re-analyzed using the Knapp-Hartung method and Bayesian random-effects. Eleven observational studies were included. ACE inhibitors increased the risk of acute pancreatitis (OR 1.33; 95% CI 1.12-1.58; I² = 93%), whereas ARBs did not (OR 0.82; 95% CI 0.80-0.83; I² = 0%). Few studies presented disaggregated results for the sensitivity analyses, but most estimates were consistent with the initial findings. The Knapp-Hartung method and Bayesian random-effects meta-analyses yielded similar results. The incidence rate of acute pancreatitis among patients treated with ACE inhibitors was 0.98 cases per 1,000 person-years, while for ARBs it was 0.71 cases per 1,000 person-years. These results suggest that ACE inhibitors increase the risk of acute pancreatitis, in contrast to the effect of ARBs. Healthcare professionals should be aware of the potential risk of pancreatitis when managing patients receiving these medications.

## Introduction and background

Acute pancreatitis is the sudden inflammation of the pancreas and is one of the most common causes of hospitalization among gastrointestinal disorders [1]. Patients may initially experience mild discomfort such as nausea, vomiting, diarrhea, and pyrexia, but the condition can progress to serious complications, including pancreatic necrosis, pseudocyst formation, mesenteric or splenic venous thrombosis, hemorrhage, and multiorgan failure [1]. The incidence of acute pancreatitis has been rising in Western countries over the past decades [2]. The mortality rate ranges from 3% in patients with mild disease to as high as 20% in severe cases with necrosis [3]. Coexisting type 2 diabetes increases the risk of complications and death [3]. The most common risk factors for acute pancreatitis are gallstone disease, excessive alcohol consumption, and hypertriglyceridemia [1]. However, some drugs are also known to cause this condition [4].

Acute pancreatitis induced by drugs acting on the Renin-Angiotensin-Aldosterone System (RAAS) is rare, but cases of patients developing this adverse reaction, even under chronic treatment, have been reported in the literature [5-9]. Observational, comparative studies evaluating this relationship use different methodological approaches and report conflicting results. Some suggest that angiotensin-converting enzyme (ACE) inhibitors may be associated with at least a moderate increased risk of acute pancreatitis [10-14]. In contrast, angiotensin II receptor blockers (ARBs) appear not to alter, or may even reduce, the risk of acute pancreatitis among users [15-17].

ACE inhibitors and ARBs are among the major drug classes with robust evidence demonstrating a reduction in cardiovascular events related to arterial hypertension, heart failure, and acute coronary syndromes, thereby improving quality of life and survival [18-20]. Given that these medications are commonly prescribed and used in long-term treatment regimens, often for chronic conditions, it is important from a public health perspective to evaluate the consistency of findings from observational studies investigating their potential association with acute pancreatitis. The aim of this systematic review and meta-analysis is to assess the risk of acute pancreatitis associated with RAAS inhibitors, specifically ACE inhibitors and ARBs.

This study was previously presented in part as a poster (EPH8) at the ISPOR Europe 2024 conference (Barcelona, Spain; November 2024). A conference abstract related to this presentation was published in Value in Health. The work was recognized as a Research Presentation Award Finalist (Top 5%) by the International Society for Pharmacoeconomics and Outcomes Research (ISPOR).

## Review

Methods

The protocol for this study was registered at PROSPERO (CRD42024593796). This systematic review and meta-analysis was conducted and reported according to the Centre for Reviews and Dissemination’s (CRD) guidance and the “Preferred Reporting Items for Systematic Reviews and Meta-Analyses (PRISMA) 2020” [21,22]. The PRISMA Checklist is presented in Appendix 1.

Literature Search

PubMed and Embase were searched from their inception until April 10, 2025, to identify studies evaluating the risk of acute pancreatitis associated with ACE inhibitors and/or ARBs. Search terms related to the adverse event and the treatments were combined. Bibliographic reference lists of all relevant studies were hand-searched to identify additional eligible studies. Only literature published in English was considered for inclusion. More details of the search strategy are shown in Appendix 2.

Eligibility Criteria

The inclusion criteria for studies were as follows: (i) comparative observational studies (case-control or cohort); (ii) patients of all ages and genders; (iii) comparison of RAAS inhibitors, ACE inhibitors, or ARBs with a non-user or active control; and (iv) provision of effect estimates on the risk of acute pancreatitis (relative risk (RR), odds ratio (OR), or hazard ratio (HR)). Only peer-reviewed full papers were considered. Basic science studies, reviews, case reports, and studies without a comparison group were excluded.

Study Selection

Two researchers independently screened the titles and abstracts by hand and selected full articles for inclusion. Disagreements were resolved by discussion and consensus with a third researcher.

Data Collection

The following data were extracted from each study: reference, study design, population, intervention, comparator, outcomes, and results. Data were extracted independently by two researchers using a pre-developed form.

Risk of Bias Assessment

The Risk of Bias in Non-randomized Studies of Interventions (ROBINS-I) tool, developed by the Cochrane Collaboration, was used to assess the risk of bias in non-randomized studies [23,24]. Each assessment was graded into one of five categories: low risk of bias, moderate risk of bias, serious risk of bias, critical risk of bias, or no information.

Data Analysis and Data Synthesis

A meta-analysis was performed by pooling rate ratios (RRs) with their 95% CIs, using the DerSimonian and Laird random-effects model [25]. The most adjusted effect size estimate was used when more than one estimate was available. For case-control studies, risk estimates from the exposure window defined as “current use” were used in the meta-analysis. Analyses were stratified according to different comparators. The I² statistic was used to assess heterogeneity between studies, with I² >50% considered indicative of substantial heterogeneity [26]. Publication bias was assessed using funnel plots [27].

A sensitivity analysis was conducted to explore the influence of study design (case-control vs. cohort), mean treatment duration (<6 or ≥6 months), dose (daily defined dose (DDD)), age (<65 or ≥65 years), active substance, and Risk of Bias scores on the results. The robustness of the initial findings was also assessed using two methods to recalculate risks: (a) the Knapp-Hartung method in combination with the Paule-Mandel estimator for between-study variance [28]; and (b) a Bayesian random-effects meta-analysis [29]. A 95% prediction interval (PrI) was also estimated.

Pooled estimates of incidence rates of acute pancreatitis among patients treated with ACE inhibitors and ARBs in cohort studies were calculated using a random-effects model. Statistical analyses were performed using R software (version 4.4.0).

Results

**Figure 1:**
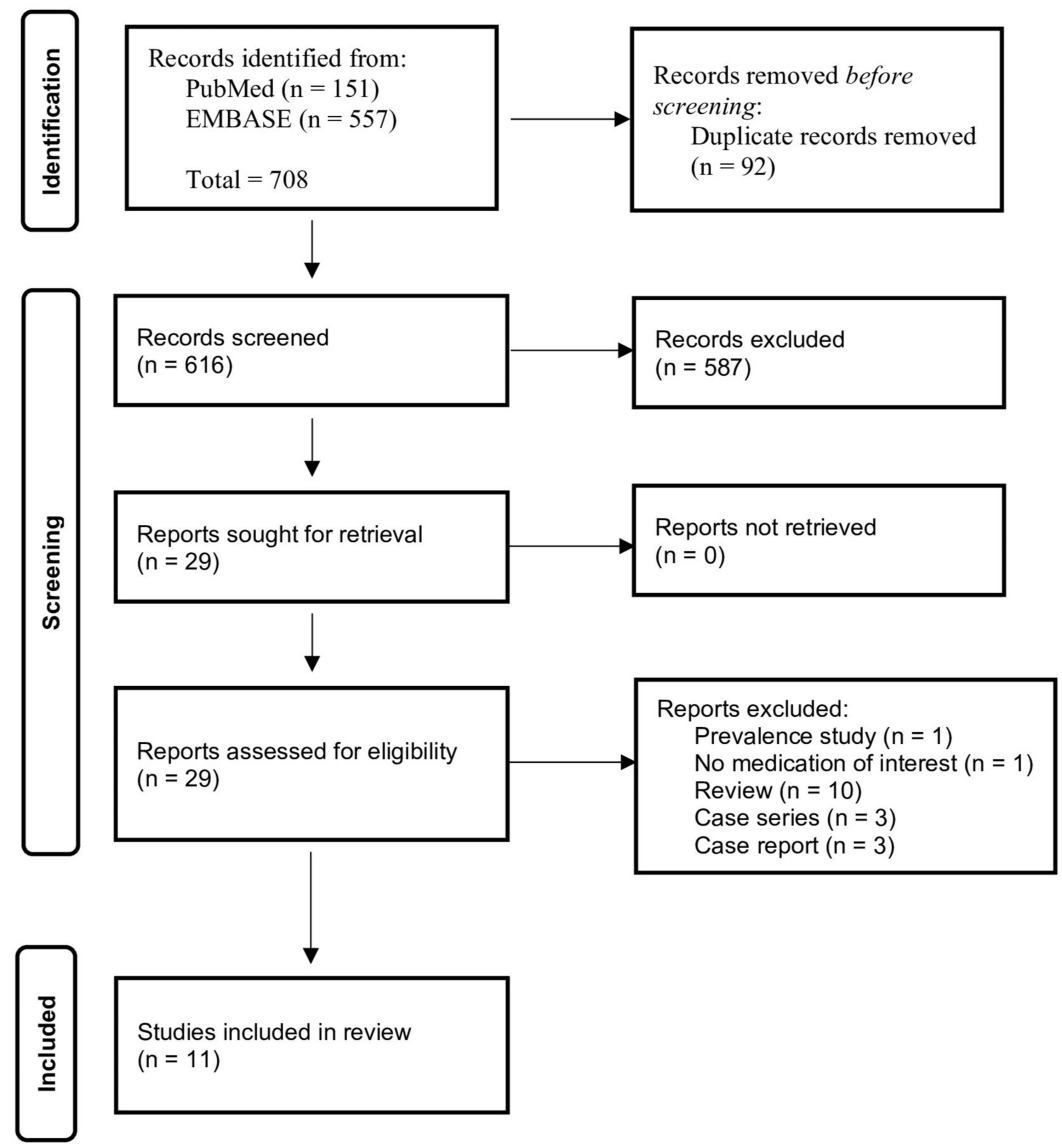
PRISMA flowchart. PRISMA: Preferred Reporting Items for Systematic Reviews and Meta-Analyses.

A total of 708 references were identified, of which 92 were duplicates. After reviewing titles and abstracts, a further 616 references were excluded. Lastly, 29 references were assessed for eligibility. Of these, 11 observational studies (7 case-control and 4 retrospective cohorts) were included [10-17,30-32]. No additional studies were identified by hand-searching reference lists. Full details of the PRISMA flow diagram are shown in Figure 1.

Characteristics of the Studies

Three studies were conducted in the United Kingdom, one in Germany, one in Sweden, one in Canada, one in Finland, one in Taiwan, one in the USA, and two were multinational. The sample sizes ranged from 852 to 2,660,521 patients. The median follow-up time among cohort studies varied from 0.7 to 5.8 years. The mean age of patients ranged between 49 and 77 years, and the proportion of female patients ranged between 39% and 62%; however, not all studies reported this information. The length of “current exposure” periods before event indexing ranged from 0-7 to 0-180 days. All studies assessed the risk of acute pancreatitis associated with ACE inhibitors or ARBs as drug classes, except one, which specifically evaluated losartan [17]. In cohort studies, comparators were dihydropyridine calcium channel blockers (dhCCBs) (2 studies), warfarin (1 study), and ACE inhibitors/ARBs (1 study). The number of covariates considered in the analyses ranged from 2 to 31, with one study not disclosing the exact number [30]. Three studies used propensity scores to match participants. The characteristics of the studies are summarized in Table 1.

**Table 1:** Characteristics of observational studies evaluating the risk of acute pancreatitis associated with ACE inhibitors and ARBs. ACE: Angiotensin-converting enzyme; ACEi: Angiotensin-converting enzyme inhibitor; ARB: Angiotensin II receptor blocker; BP: Bisphosphonates; CCB: Calcium channel blocker; dhCCB: Dihydropyridine calcium channel blocker; ERCP: Endoscopic retrograde cholangiopancreatography; HbA1c: Glycated hemoglobin; ICD: International Classification of Diseases; IV: Intravenous; MPR: Medication possession ratio; NR: Not reported; NSAIDs: Non-steroidal anti-inflammatory drugs. * Presented results for length of treatment use, which were included in the sensitivity analysis.

Reference	Study design	Data sources (Country)	Follow-up (mean) / Exposure window (days, interval)	Population	Intervention / Cases	Comparator / Control	Confounding adjustment (number of covariates)	Mean age (years)	Female (%)
Lancashire RJ et al., (2003) [32]	Case-control	General Practitioner Research Database (United Kingdom)	1-90 days (recent)	Total population includes 3-4 million from all areas of the UK. Events recorded include all hospitalizations and all instances in which treatment was withdrawn.	Patients diagnosed with acute pancreatitis (ICD 577.0), from 03/1989 to 11/1998 (n = 3673)	Patients without a diagnosis of acute pancreatitis at the time the case was diagnosed. Three controls for each case, matched for age, sex, and general practice	Age, sex, general practice (3)	NR	NR
Cheng RM et al., (2003) [10]	Retrospective cohort	Ontario Drug Benefit (ODB) Program, Canadian Institute for Health Information Discharge Abstract Database, Ontario Health Insurance Plan, Ontario Registered Persons Database (Canada)	ACE inhibitors = 1.47 years (535 ± 528 days); Warfarin = 1.12 years (409 ± 467 days)	Ontario residents (>1.4 million), aged >65 years, dispensed ACE inhibitors, warfarin, or dihydropyridine calcium channel blockers, from 01/01/1994 to 31/03/2000	ACE inhibitors (n = 174,824)	Warfarin (n = 40,057)	Age, gender, lipid disorders, thyroid disease, hepatitis B, use of furosemide/hydrochlorothiazide, estrogens, NSAIDs (8)	ACEi (76.6), Warfarin (77.5)	ACEi (58.6), Warfarin (59.6)
Eland IA et al., (2006) [14]	Case-control	European case-control study on drug-induced acute pancreatitis (EDIP): Denmark, Netherlands, Italy, Sweden (Multinational)	0-7 days (current)*	Patients aged 40-85 years, hospitalized for acute pancreatitis between 01/10/1994 and 31/12/1998	Acute pancreatitis (n = 724; ACE inhibitors = 83; ARBs = 8)	Controls matched to cases on gender and age (±5 years). In Italy/Netherlands: 1 control per case; in Denmark/Sweden: 2-4 controls per case (Total = 1791; ACE inhibitors = 117; ARBs = 22)	Age, gender, antihypertensives, alcohol use, smoking, BMI, pancreatic disease, biliary disease, diabetes, heart failure (10)	Cases (62.7), Controls (60.6)	Cases (41.7), Controls (48.1)
Sjöberg Bexelius T et al., (2009) [15]	Case-control	The Health Improvement Network (THIN) database (United Kingdom)	0-14 days (current)*	Registered ≥2 years with GP, diagnosed with hypertension, aged 40-80 years between 01/01/1996 and 30/09/2005	Acute pancreatitis (n = 265; ARBs = 22; ACE inhibitors = 86)	Randomly selected controls frequency-matched by sex, age (±1 year), calendar year (n = 2000)	Sex, age, year, smoking, alcohol, BMI, use of antihypertensives, GP visits (13)	NR	Cases (52.4), Controls (52.1)
Douros A et al., (2013) [13]	Case-control	Hospital-based Berlin case-control surveillance study FAKOS (51 hospitals, Germany)	0-7 days (current)	Pancreatic toxicity study covering >180 departments across 51 hospitals (10/2002-12/2011), source population of 2.8 million	Idiopathic acute pancreatitis within 6 months (n = 102; ACE inhibitors = 22)	Controls representative of hospital drug use, 7:1 ratio (n = 750; ACE inhibitors = 127)	Age, sex (2)	Cases (49.3), Controls (NR)	Cases (48), Controls (NR)
Bexelius TS et al., (2017) [16]	Case-control	Swedish Patient Register, Prescribed Drug Register, Cancer Register, Causes of Death Register, National Education Register (Sweden)	0-114 days (current)*	Swedish residents aged 40-84 years during 01/01/2006-31/12/2008	Acute pancreatitis (n = 61,601; ARBs = 548; ACE inhibitors = 987)	General population controls matched by age, sex, year (n = 61,637; ARBs = 4715; ACE inhibitors = 6067)	Sex, age, year, education, alcohol-related disease, gallstones, COPD, diabetes, CVD, medications (12)	NR	Cases (45), Controls (45)
Kuoppala J et al., (2017) [11]	Case-control	National Institute for Health and Welfare, Social Insurance Institution (SII) Prescription Register, Register on Special Reimbursement Rights for Chronic Diseases (Finland)	0-180 days (current)	Subjects ≥18 years, first non-biliary, non-alcohol-induced acute pancreatitis during 01/01/2008-31/12/2010	Acute pancreatitis (n = 4966; ACE inhibitors = 1276)	5 controls matched for sex, birth year per patient (n = 24,788; ACE inhibitors = 3946)	Sex, age, ACEi use, gallstones, alcohol disease, chronic pancreatic insufficiency, GI disease, diabetes, hypothyroidism, statins, fibrates, ezetimibe, ERCP, cholecystectomy, institutional care (15)	59	Cases (41), Controls (41)
Lai SW et al., (2017) [17]	Case-control	Taiwan National Health Insurance Program (Taiwan)	0-7 days (current)	Database covering 99% of 23M citizens, hypertensive subjects aged 20-84 years, 2000-2011	First episode of acute pancreatitis (n = 1449; Losartan = 52)	Hypertensive controls without pancreatitis, randomly selected (n = 2479; Losartan = 90)	Age, sex, antihypertensives, alcohol-related disease, biliary stones, CKD, hepatitis B/C, hyperparathyroidism, hypertriglyceridemia (10)	Cases (59.4), Controls (59.0)	Cases (62.1), Controls (61.9)
Chen R et al., (2021) [30]	Retrospective cohort	IBM MarketScan (USA), Optum Clinformatics (USA), Korea National Health Insurance (Korea), IQVIA Germany, Columbia University (USA), Optum EHR (USA) (Multinational)	NR	Patients initiating monotherapy with ACE inhibitors or ARBs	ACE inhibitors (n = 2,297,881); ARBs (n = 673,938)	ARBs (n = 673,938); ACE inhibitors (n = 2,297,881)	Propensity scores: demographics, diagnoses, drug exposures, procedures, comorbidities, risk scores (NR)	NR	ACEi (38.7), ARBs (39.2)
Rouette J et al., (2022) [12]	Retrospective cohort	Clinical Practice Research Datalink (CPRD), Hospital Episode Statistics (HES), Office for National Statistics (ONS) (UK)	Cohort 1: 0.8 years; Cohort 2: 0.7 years	Two new-user, active comparator cohorts (1998-2018; follow-up until 2019)	Cohort 1: ACE inhibitors = 304,083; Cohort 2: ARBs = 29,160	Cohort 1: dhCCBs (n = 283,403); Cohort 2: dhCCBs (n = 33,721)	Propensity scores: age, sex, BMI, smoking, alcohol, CVD, gallstones, prior pancreatitis, drugs linked to pancreatitis (statins, fibrates, valproic acid, diuretics, antidiabetics), cancer screenings, vaccines (31)	Cohort 1: ACEi (62.1), dhCCBs (62.5); Cohort 2: ARBs (62.6), dhCCBs (63.6)	Cohort 1: ACEi (46.3), dhCCBs (48.5); Cohort 2: ARBs (52.5), dhCCBs (53.5)
Krishnan A et al., (2023) [31]	Retrospective cohort	TriNetX database (USA)	ACEi = 5.8 years; ARBs = 5.3 years; dhCCBs = 4.9 years	9,929,954 new users of ACEi, ARBs, dhCCBs, 2010-2021	ACEi = 3,040,83; ARBs = 2,660,521	dhCCBs matched 1:1 with both ACEi and ARB cohorts	Propensity scores: demographics, BMI, smoking, alcohol, comorbidities, HbA1c, BP, other CV and antidiabetic drugs (9)	NR	NR

Risk of Bias

**Figure 2:**
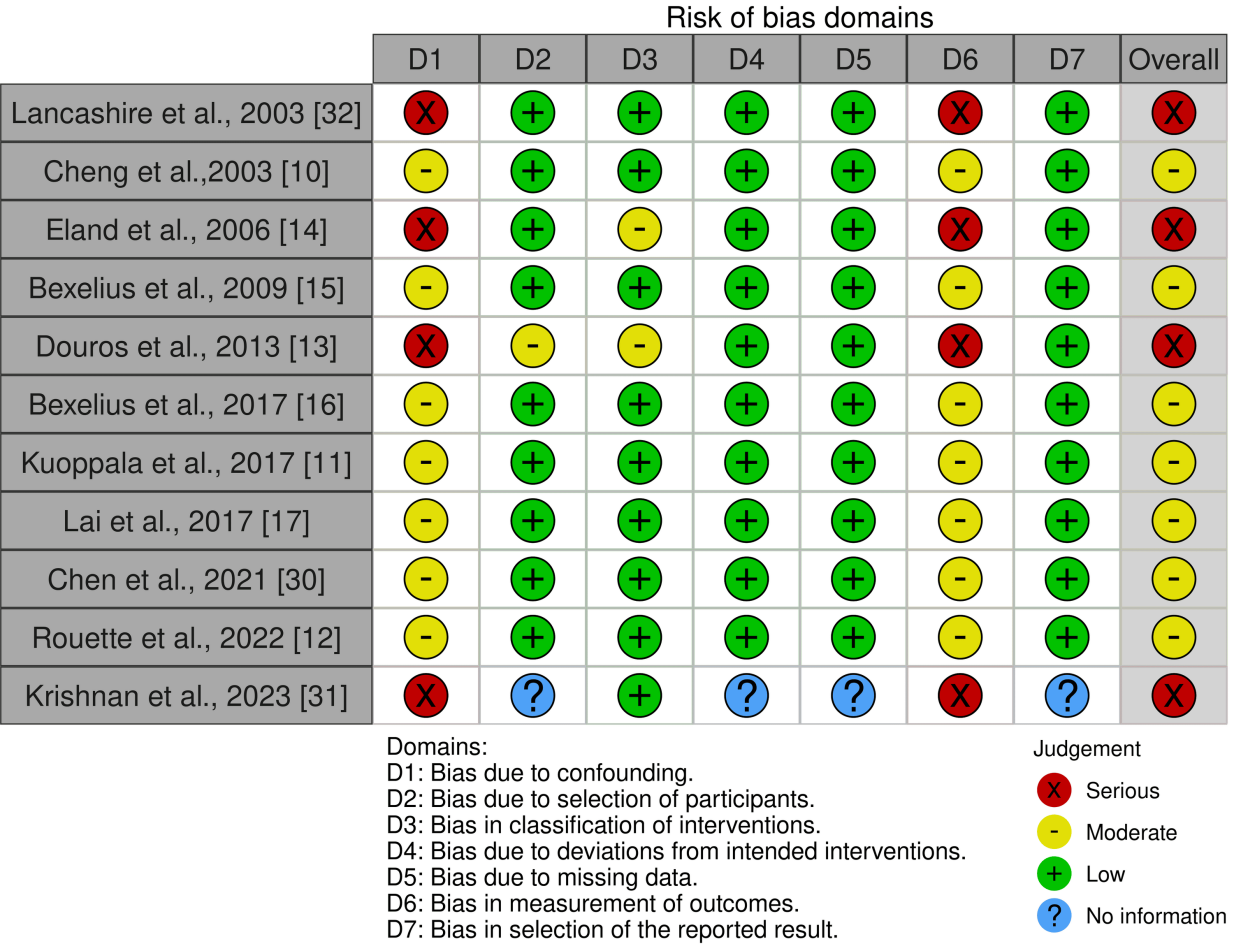
Risk of bias assessment using the ROBINS-I tool. Figure generated with the robvis tool [22]. ROBINS-I: Risk of Bias In non-randomized Studies of Interventions.

Seven studies were assessed as having a moderate risk of bias, and four as having a serious risk of bias. Data on methods were not always clear regarding how confounding was avoided. Recall or information bias was considered a limitation in case-control studies in which information on drug intake, demographics, medical history, and other possible risk factors was determined retrospectively through interviews or questionnaires. In addition, outcome assessors could have been aware of the intervention received. However, it was unclear how this may have influenced outcome measurement. As the article by Krishnan A et al. (2023) is an abstract publication, there was insufficient information to make a judgment about the risk of bias [31]. The results of the risk of bias assessment are illustrated in Figure 2.

Risk of Acute Pancreatitis

**Figure 3:**
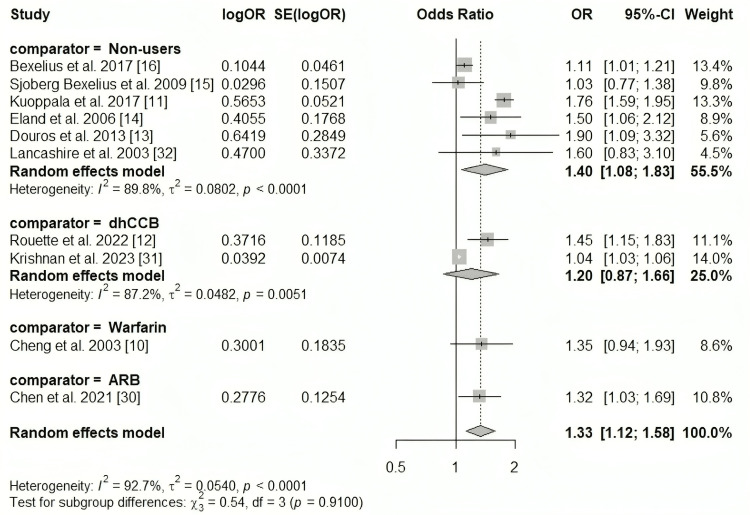
Meta-analysis of the risk of acute pancreatitis associated with ACE inhibitors. ACE: Angiotensin-converting enzyme; ARB: Angiotensin II receptor blocker; dhCCB: Dihydropyridine calcium channel blocker; OR: Odds ratio; SE: Standard error.

Overall, ACE inhibitors increased the risk of acute pancreatitis (OR 1.33; 95% CI 1.12-1.58; I² = 93%). Results were similar when the comparison was restricted to non-users (OR 1.40; 95% CI 1.08-1.83; I² = 90%), but no statistically significant differences were observed when comparing ACE inhibitors with active treatments. A forest plot illustrating the risk estimates for ACE inhibitors is shown in Figure 3.

**Figure 4:**
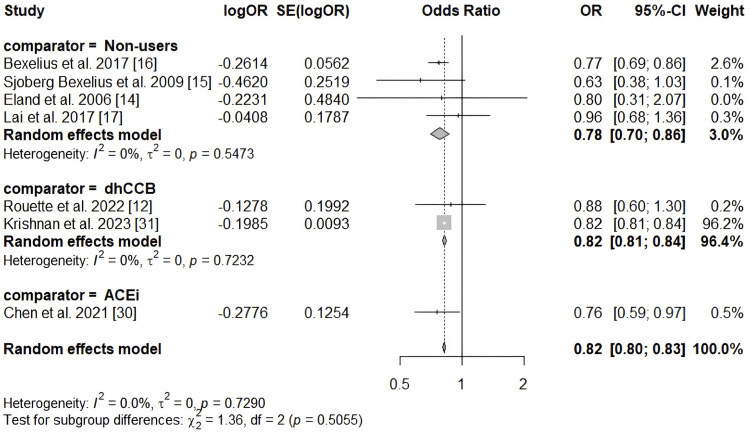
Meta-analysis of the risk of acute pancreatitis associated with ARBs. ACEi: Angiotensin-converting enzyme inhibitor; ARB: Angiotensin II receptor blocker; dhCCB: Dihydropyridine calcium channel blocker; OR: Odds ratio; SE: Standard error.

ARBs were associated with a reduced risk of acute pancreatitis (OR 0.82; 95% CI 0.80-0.83; I² = 0%). The results did not vary according to the comparator used. A forest plot illustrating the risk estimates for ARBs is shown in Figure 4.

Sensitivity Analysis

ACE inhibitors were associated with an increased risk of acute pancreatitis in case-control studies (OR 1.39; 95% CI 1.10-1.74; I² = 88%), but not in cohort studies. The risk associated with ARBs did not differ by study design (case-control vs. cohort). Stratification by mean treatment duration (<6 or ≥6 months) did not alter the initial results for either ACE inhibitors or ARBs.

ACE inhibitors were associated with an increased risk of acute pancreatitis among patients younger than 65 years, whereas no differences were observed in other subgroup analyses. Stratification by mean daily dose showed an increased risk for >2 DDD (OR 1.75; 95% CI 1.57-1.95; I² = 0%); no differences were observed for other doses. Only one study stratified results by mean daily dose for ARBs, without statistically significant findings [15].

Risk remained increased when results were disaggregated by ACE inhibitor active substance; no risk differences were observed for individual ARBs. Stratification by Risk of Bias scores did not materially change the initial estimates. Subgroup analysis results are detailed in Table 2.

**Table 2:** Meta-analysis of the risk of acute pancreatitis associated with ACE inhibitors and ARBs. ACE: Angiotensin-converting enzyme; ARB: Angiotensin II receptor blocker; DDD: Defined daily dose; NR: Not reported.

Category	Studies (n)	OR	95% CI	I²
ACE inhibitors				
Overall	10	1.33	1.12-1.58	93%
Design				
Case-control	7	1.4	1.10-1.79	88%
Cohort	3	1.23	0.95-1.59	80%
Mean follow-up				
<6 months	1	3.2	2.90-3.53	-
≥6 months	4	1.26	1.00-1.59	75%
Age				
<65 years	4	1.7	1.65-1.85	0%
≥65 years	1	1.35	0.94-1.93	-
Active substance				
Enalapril	3	1.63	1.39-1.90	0%
Lisinopril	3	1.71	1.39-2.09	2%
Perindopril	2	1.6	1.28-2.01	0%
Ramipril	3	1.69	1.38-2.08	36%
Captopril	1	3.1	1.18-8.15	-
Dose				
<1 DDD	2	1.61	1.33-1.95	15%
1-2 DDD	2	1.55	1.38-1.75	39%
>2 DDD	2	1.75	1.57-1.95	0%
Risk of bias				
Moderate	6	1.32	1.06-1.65	89%
Serious	4	1.37	0.99-1.89	71%
ARBs				
Overall	7	0.82	0.80-0.83	0%
Design				
Case-control	5	0.78	0.71-0.85	0%
Cohort	2	0.82	0.81-0.84	0%
Mean follow-up				
<6 months	2	0.68	0.47-0.99	15%
≥6 months	4	0.82	0.80-0.83	0%
Age				
<65 years	3	0.91	0.71-1.18	0%
≥65 years	0	-	-	-
Active substance				
Candesartan	1	1.27	0.69-2.34	-
Irbesartan	1	0.53	0.21-1.35	-
Losartan	2	0.97	0.72-1.32	0%
Valsartan	1	0.29	0.04-2.04	-
Dose	NR	-	-	-
Risk of bias				
Moderate	5	0.78	0.71-0.86	0%
Serious	2	0.82	0.81-0.84	0%

An increased risk of acute pancreatitis associated with ACE inhibitors was also observed using the Knapp-Hartung method (OR 1.32; 95% CI 1.13-1.54; I² = 93%). The 95% PrI was estimated as RR: 0.87-1.99. ARBs maintained a reduced risk when the Knapp-Hartung method was employed (OR 0.82; 95% CI 0.80-0.83; I² = 0%); the 95% PrI was 0.80-0.84. Bayesian meta-analysis also showed an increased risk with ACE inhibitors (OR 1.236; 95% CrI 1.002-1.589), but not with ARBs (OR 0.807; 95% CrI 0.628-1.032). Details of recalculated risks are provided in Appendices 3-6.

Incidence Rate

The incidence rate of acute pancreatitis among patients treated with ACE inhibitors was estimated at 0.98 cases per 1000 person-years. For patients treated with ARBs, the incidence rate was 0.71 cases per 1000 person-years. Detailed results of incidence analyses are presented in Appendices 7-8.

Publication Bias

No significant asymmetry was identified in the funnel plots of either meta-analysis, but the low number of studies limited these assessments. Details of publication bias analyses are provided in Appendices 9-10.

Discussion

ACE inhibitors and ARBs are extensively prescribed worldwide to treat cardiovascular and renal diseases [33,34]. Although pharmacological interventions are not the most frequent cause of acute pancreatitis, this adverse reaction can have severe and potentially fatal consequences for patients [4]. The risk of acute pancreatitis induced by RAAS inhibitors has been continuously investigated through observational analytical studies published over the years [5-7,35,36]. Therefore, it was relevant to assess the consistency of those results. To our knowledge, this is the first systematic review and meta-analysis aimed at characterizing the risk of acute pancreatitis associated with RAAS inhibitors. Our findings suggest a possible differential effect: ACE inhibitors may increase the risk of acute pancreatitis, while ARBs may have a protective effect. This contrasts with the losartan SmPC, in which acute pancreatitis is cited as a possible adverse reaction, though with an unknown frequency [37].

The mechanisms underlying these effects remain incompletely understood. Inhibition of ACE reduces the degradation of bradykinin into inactive metabolites [38]. Accumulation of bradykinin may increase pancreatic vascular permeability, promote oedema, and inhibit the release of pancreatic enzymes, ultimately leading to inflammation and tissue damage, as suggested by preclinical in vivo studies [12,39]. The effect of ARBs may result from their interference with the renin-angiotensin system in the pancreas, which plays an important role in regulating pancreatic duct and digestive enzyme secretion [12]. Animal models indicate that ARBs may reduce pancreatic inflammation and necrosis by inhibiting digestive enzyme secretion, which could explain a protective association against acute pancreatitis [40]. These biological mechanisms appear to support the results observed in this meta-analysis. However, these hypotheses require further validation in clinical settings. The observational studies included present varying demographic characteristics and methodologies, which complicates direct comparisons.

Gender distribution of the study populations also varied. Evidence indicates that females with acute pancreatitis have significantly better clinical outcomes than males, including a lower likelihood of mortality [41]. However, it was not possible to conduct a sensitivity analysis because most studies did not report sex-disaggregated results. The mean age of participants also varied substantially. Both the incidence and severity of acute pancreatitis are known to increase with age [42]. Our analysis showed that the RR associated with ACE inhibitors was significantly elevated in patients younger than 65 years, with no significant association observed in older age groups. Although an age-stratified analysis (<65 or ≥65 years) was performed, the interpretability of the findings is limited due to incomplete reporting of age-specific data in some studies. This counterintuitive result may reflect differences in background risk: in older individuals, pancreatitis may more often arise from other etiologies such as gallstones, alcohol use, or metabolic disorders, thereby attenuating the observable effect of ACE inhibitors. In younger patients, by contrast, the drug’s contribution to risk may be more pronounced.

The control groups used by the observational studies also differed. Non-use of ACE inhibitors/ARBs was the most common comparator group, but some studies used active controls. Dihydropyridine calcium channel blockers (dhCCBs) were used to control for background disease in some studies, and warfarin was used in one study to control for baseline incidence of acute pancreatitis [12,15,31]. One study compared ACE inhibitors with ARBs to reduce the risk of confounding by indication. The use of different comparator groups introduces heterogeneity, as baseline risk may vary between non-users and users of other cardiovascular drugs. The risk of acute pancreatitis associated with ARBs did not change when the analysis was stratified by different comparators. For ACE inhibitors, the results became statistically non-significant when the analysis was restricted to dhCCBs and warfarin as comparators, but only two and one study, respectively, were available. Therefore, these findings should be interpreted cautiously, as the small number of studies limits the statistical power of these subgroup analyses.

There are also significant differences between studies regarding the length of treatment use. Follow-up varied from 0.7 to 5.8 years among cohort studies, and only three case-control studies presented risk estimates according to different treatment durations. Although a protective effect against acute pancreatitis has been linked to longer treatment durations with ARBs, our sensitivity analyses did not show a consistent relationship between treatment duration and risk for either drug class [16]. A similar limitation precluded a thorough analysis of risk according to different doses. Only two studies reported results for different ACE inhibitor DDD categories, without significant risk differences [11,14]. Although Sjöberg Bexelius T et al. (2009) reported results for ARBs according to low/medium and high doses, these categories could not be precisely related to DDD, yet the study was considered in the analysis [15]. As a result, the limited number of studies reporting dose and duration data hinders a robust assessment of any dose-response or time-related trends.

Analysis according to active substances did not reveal differences for ACE inhibitors. Regarding ARBs, data are scarce, as only one study reported results for most of the molecules, with few events occurring [12]. The exception is losartan, with data reported by two studies, although no association with acute pancreatitis was observed. However, as most of the studies did not provide risk estimates by active substance, this remains an important gap that future studies should address.

The stratification of results according to different study designs did not significantly alter the initial findings, except in the cohort subgroup for ACE inhibitors, which returned a statistically non-significant risk. It should be noted that retrospective cohort studies selected different active comparators (dhCCBs or warfarin) [10,12]. Although none of these drug classes have been associated with acute pancreatitis, the baseline risk of this event may differ when compared with non-users. This variation in study design and comparator selection may partly explain the inconsistency of findings across study subtypes.

The incidence rate of acute pancreatitis was higher with ACE inhibitors. Although the event is rare, ACE inhibitors presented an excess of 0.3 cases per 1000 person-years compared with ARBs. Nonetheless, drug-induced acute pancreatitis is rare, and cases associated with ACE inhibitors are usually described in the literature as case reports, which hampers identification in clinical trials [5-7,43,44].

Additional limitations should be considered. First, case-control studies differed in how they defined the exposure window for “current/recent use,” which ranged from 7 to 180 days. Although some studies conducted risk analyses according to different windows (current vs. past use), these disparities highlight heterogeneity in how cases and controls were matched. Second, between-study heterogeneity was identified, particularly in the ACE inhibitor meta-analysis. This was expected since only observational studies were included. As frequentist methods may underestimate heterogeneity, additional analyses were conducted using the Knapp-Hartung method (combined with the Paule-Mandel estimator), as recommended by the Cochrane Collaboration, and the Bayesian random-effects method [28]. Third, studies showed some risk of bias, with ratings of “moderate” or “serious.” The number of covariates used for adjustment and the approaches to control confounders varied considerably. For example, Douros A et al. (2013) adjusted for two covariates, Rouette J et al. (2022) for 31, and Chen R et al. (2021) stated that “tens of thousands” of covariates were considered but did not report an exact number [12,13,30]. Furthermore, the three most recent studies used propensity scores to match participants on potential confounders [12,30,31]. This variation in statistical adjustment introduces inconsistency in the reliability of effect estimates, particularly regarding confounders such as alcohol consumption, gallstones, hypertriglyceridemia, and concurrent medications like NSAIDs. Moreover, as all studies were retrospective/transversal, recall bias cannot be excluded. Fourth, only two cohort studies reported incidence rates of acute pancreatitis, preventing a meta-analysis of this important outcome.

## Conclusions

The present findings suggest an increased risk of pancreatitis associated with ACE inhibitors and a reduced risk with ARBs, although the methodological limitations inherent in observational research preclude definitive conclusions. Given the rarity but clinical relevance of drug-induced pancreatitis, clinicians should remain vigilant when prescribing RAAS inhibitors, particularly ACE inhibitors. Enhanced pharmacovigilance activities are warranted to further clarify these associations.

## Figures and Tables

**Figure 5:**
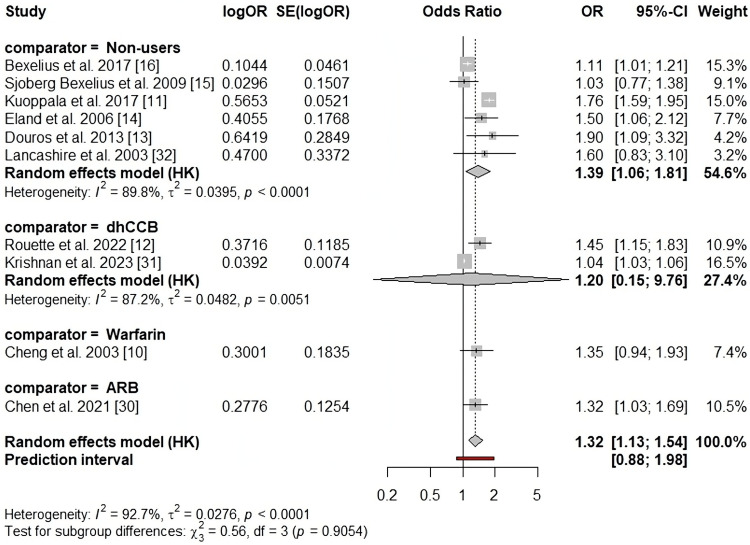
Meta-analysis of the risk of acute pancreatitis associated with ACE inhibitors using the Knapp-Hartung method. ACE: Angiotensin-converting enzyme; ARB: Angiotensin II receptor blocker; dhCCB: Dihydropyridine calcium channel blocker; OR: Odds ratio; SE: Standard error.

**Figure 6:**
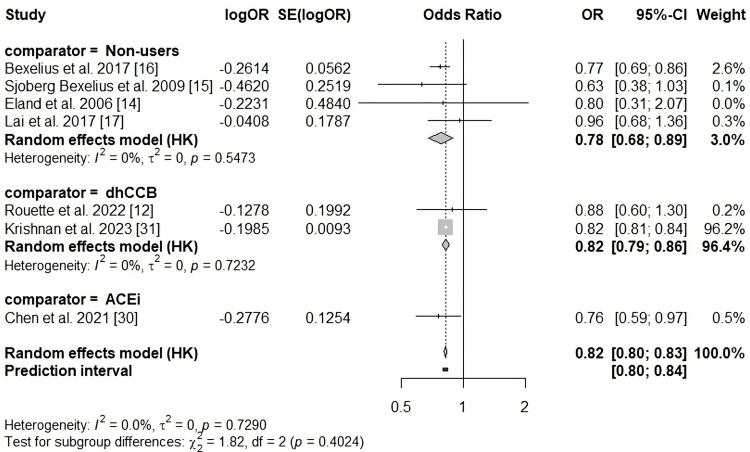
Meta-analysis of the risk of acute pancreatitis associated with ARBs using the Knapp-Hartung method. ACEi: Angiotensin-converting enzyme inhibitor; ARB: Angiotensin II receptor blocker; dhCCB: Dihydropyridine calcium channel blocker; SE: Standard error.

**Figure 7:**
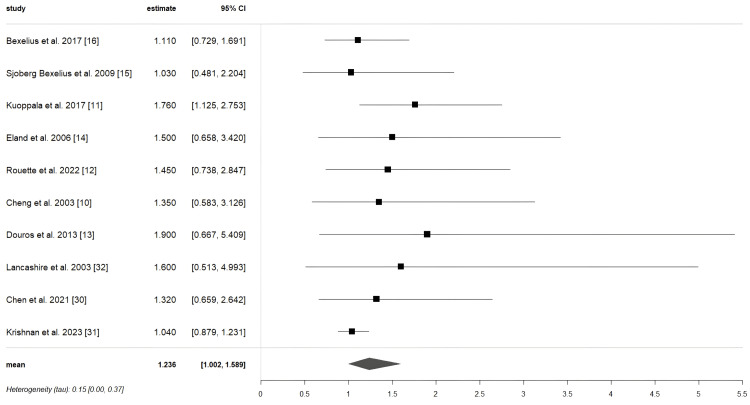
Bayesian meta-analysis of the risk of acute pancreatitis associated with ACE inhibitors. ACE: Angiotensin-converting enzyme.

**Figure 8:**
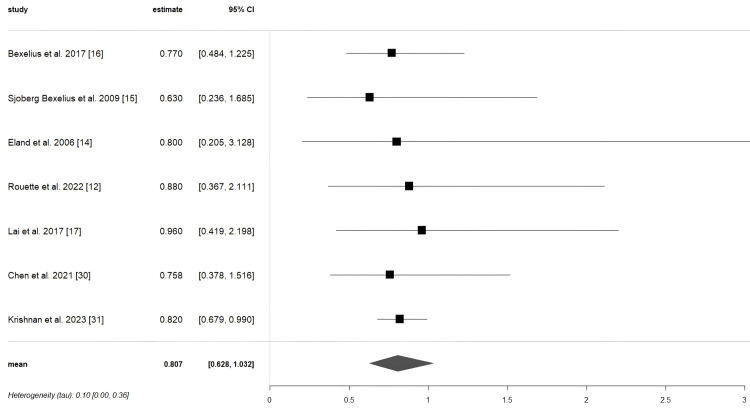
Bayesian meta-analysis of the risk of acute pancreatitis associated with ARBs. ARB: Angiotensin II receptor blocker.

**Figure 9:**
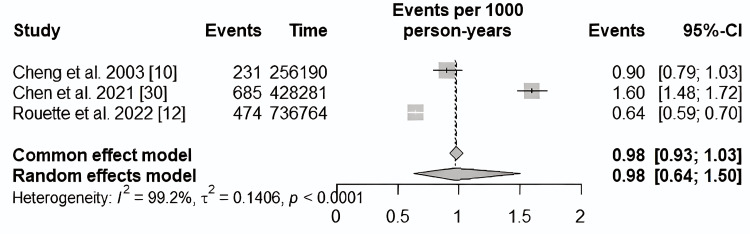
Meta-analysis of the incidence rate of acute pancreatitis among patients treated with ACE inhibitors. ACE: Angiotensin-converting enzyme.

**Figure 10:**
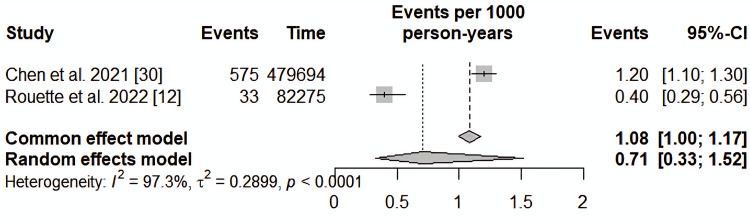
Meta-analysis of the incidence rate of acute pancreatitis among patients treated with ARBs. ARB: Angiotensin II receptor blocker.

**Figure 11:**
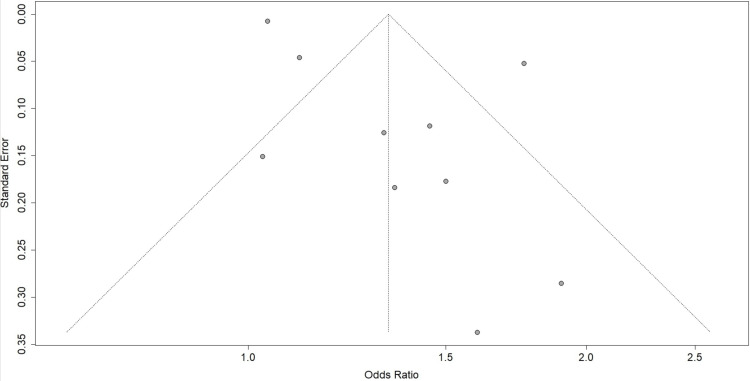
Funnel plot of studies included in the meta-analysis of the risk of acute pancreatitis associated with ACE inhibitors. ACE: Angiotensin-converting enzyme.

**Figure 12:**
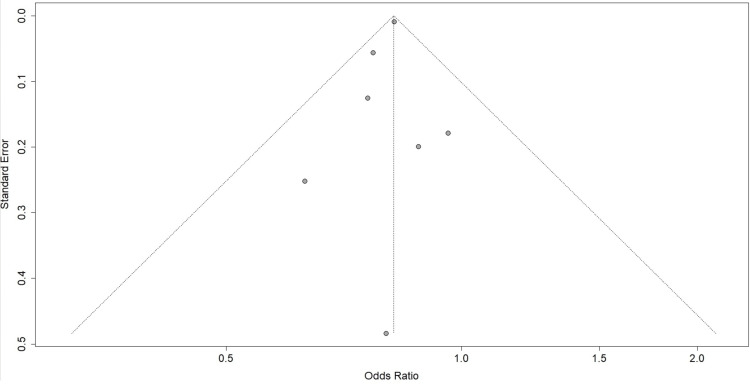
Funnel plot of studies included in the meta-analysis of the risk of acute pancreatitis associated with ARBs. ARB: Angiotensin II receptor blocker.

## Appendices

Appendix 1 

**Table 3:** PRISMA checklist. PRISMA: Preferred Reported Items for Systematic Reviews and Meta-Analysis.

Section and Topic	Item #	Checklist item	Location where item is reported
Title			
Title	1	Identify the report as a systematic review.	–
Abstract			
Abstract	2	See the PRISMA 2020 for Abstracts checklist.	Abstract section
Introduction			
Rationale	3	Describe the rationale for the review in the context of existing knowledge.	Introduction section
Objectives	4	Provide an explicit statement of the objective(s) or question(s) the review addresses.	Aim section
Methods			
Eligibility criteria	5	Specify the inclusion and exclusion criteria for the review and how studies were grouped for the syntheses.	Methods, eligibility criteria section
Information sources	6	Specify all databases, registers, websites, organizations, reference lists, and other sources searched or consulted to identify studies. Specify the date when each source was last searched or consulted.	Methods, search strategy section
Search strategy	7	Present the full search strategies for all databases, registers, and websites, including any filters and limits used.	Methods, search strategy section; Appendix B
Selection process	8	Specify the methods used to decide whether a study met the inclusion criteria of the review, including how many reviewers screened each record and report, whether they worked independently, and if applicable, details of automation tools used.	Methods, study selection section
Data collection process	9	Specify the methods used to collect data from reports, including how many reviewers collected data, whether they worked independently, any processes for obtaining or confirming data from study investigators, and if applicable, details of automation tools used.	Methods, data collection section
Data items	10a	List and define all outcomes for which data were sought. Specify whether all compatible results were sought (e.g., for all measures, time points, analyses) and, if not, the methods used to decide which results to collect.	Methods, eligibility criteria section
	10b	List and define all other variables for which data were sought (e.g., participant and intervention characteristics, funding sources). Describe any assumptions about missing or unclear information.	Methods, data collection section
Study risk of bias assessment	11	Specify the methods used to assess risk of bias in included studies, including the tool(s) used, how many reviewers assessed each study, whether they worked independently, and if applicable, details of automation tools used.	Methods, risk of bias assessment section
Effect measures	12	Specify for each outcome the effect measure(s) (e.g., risk ratio, mean difference) used in the synthesis or results presentation.	Methods, data analysis and synthesis section
Synthesis methods	13a	Describe the processes used to decide which studies were eligible for each synthesis (e.g., tabulating intervention characteristics and comparing against planned groups).	Methods, data analysis and synthesis section
	13b	Describe any methods required to prepare the data for presentation or synthesis (e.g., handling missing statistics, data conversions).	Methods, data analysis and synthesis section
	13c	Describe any methods used to tabulate or visually display results of individual studies and syntheses.	Methods, data analysis and synthesis section
	13d	Describe any methods used to synthesize results and provide a rationale for the choice(s). If meta-analysis was performed, describe the model(s), heterogeneity methods, and software used.	Methods, data analysis and synthesis section
	13e	Describe any methods used to explore possible causes of heterogeneity (e.g., subgroup analysis, meta-regression).	Methods, data analysis and synthesis section
	13f	Describe any sensitivity analyses conducted to assess robustness of the results.	Methods, data analysis and synthesis section
Reporting bias assessment	14	Describe any methods used to assess risk of bias due to missing results in a synthesis (reporting biases).	Methods, data analysis and synthesis section
Certainty assessment	15	Describe any methods used to assess certainty (or confidence) in the body of evidence.	Methods, data analysis and synthesis section
Results			
Study selection	16a	Describe the search and selection results, from records identified to studies included, ideally using a flow diagram.	Results, first paragraph; Figure 1
	16b	Cite studies that appeared to meet inclusion criteria but were excluded, and explain why.	Results, first paragraph; Figure 1
Study characteristics	17	Cite each included study and present its characteristics.	Results, characteristics of studies section; Table 1
Risk of bias in studies	18	Present risk of bias assessments for each included study.	Results, risk of bias section; Figure 2
Results of individual studies	19	Present for each study: (a) summary statistics for each group (if appropriate) and (b) an effect estimate and its precision (e.g., CI/CrI), ideally in structured tables or plots.	Results, characteristics of studies section; Table 1; Figures 3–4
Results of syntheses	20a	For each synthesis, briefly summarize study characteristics and risk of bias.	Results, risk of acute pancreatitis section; Figures 3–4
	20b	Present results of all statistical syntheses. If meta-analysis was done, report the summary estimate, its precision, and heterogeneity measures. If groups were compared, describe the direction of effect.	Results, risk of acute pancreatitis section; sensitivity analysis
	20c	Present results of investigations of possible causes of heterogeneity.	Results, risk of acute pancreatitis section; sensitivity analysis
	20d	Present results of sensitivity analyses conducted to test robustness of results.	Results, risk of acute pancreatitis section; sensitivity analysis
Reporting biases	21	Present assessments of risk of bias due to missing results (reporting biases).	Results, publication bias section
Certainty of evidence	22	Present certainty (or confidence) assessments for each outcome.	Results, risk of acute pancreatitis section; sensitivity analysis
Discussion			
Discussion	23a	Provide a general interpretation of results in the context of other evidence.	Discussion section
	23b	Discuss limitations of the evidence included.	Discussion section
	23c	Discuss limitations of the review process.	Discussion section
	23d	Discuss implications for practice, policy, and future research.	Discussion, last paragraph
Other Information			
Registration and protocol	24a	Provide registration information for the review (register name and number) or state if not registered.	NA
	24b	Indicate where the review protocol can be accessed, or state if none was prepared.	NA
	24c	Describe and explain amendments to information provided at registration or in the protocol.	NA
Support	25	Describe sources of financial/non-financial support and the role of funders/sponsors.	Declarations, funding section
Competing interests	26	Declare any competing interests of authors.	Declarations, conflicts of interest section
Availability of data, code, and other materials	27	Report which of the following are publicly available and where: data collection forms, extracted data, analytic code, or other materials.	Available upon request from authors

Appendix 2 

**Table 4:** Literature search strategies. The search covered the period from database inception to April 10, 2025.

Bibliographic database: PubMed (https://pubmed.ncbi.nlm.nih.gov/)		
Search	Equation	Results
#1	angiotensin-converting enzyme inhibitors OR angiotensin-converting enzyme inhibitors [MeSH Terms]	60,738
#2	captopril OR cilazapril OR enalapril OR lisinopril OR perindopril OR ramipril OR trandolapril OR fosinopril OR imidapril OR quinapril OR zofenopril	30,826
#3	#1 OR #2	66,092
#4	angiotensin receptor antagonists OR angiotensin receptor antagonists [MeSH Terms]	31,789
#5	candesartan OR irbesartan OR losartan OR olmesartan OR telmisartan OR valsartan OR eprosartan OR azilsartan	25,604
#6	#4 OR #5	43,264
#7	#3 OR #6	92,625
#8	acute pancreatitis OR acute pancreatitis [MeSH Terms]	82,664
#9	#7 AND #8	151
Bibliographic database: EMBASE (https://www.embase.com)		
Search	Equation	Results
#1	angiotensin converting' AND ('enzyme'/exp OR enzyme) AND ('inhibitors'/exp OR inhibitors)	30,451
#2	captopril'/exp OR captopril OR 'cilazapril'/exp OR cilazapril OR 'enalapril'/exp OR enalapril OR 'lisinopril'/exp OR lisinopril OR 'perindopril'/exp OR perindopril OR 'ramipril'/exp OR ramipril OR 'trandolapril'/exp OR trandolapril OR 'fosinopril'/exp OR fosinopril OR 'imidapril'/exp OR imidapril OR 'quinapril'/exp OR quinapril OR 'zofenopril'/exp OR zofenopril	100,078
#3	('angiotensin'/exp OR angiotensin) AND ('receptor'/exp OR receptor) AND antagonists	12,048
#4	candesartan'/exp OR candesartan OR 'irbesartan'/exp OR irbesartan OR 'losartan'/exp OR losartan OR 'olmesartan'/exp OR olmesartan OR 'telmisartan'/exp OR telmisartan OR 'valsartan'/exp OR valsartan OR 'eprosartan'/exp OR eprosartan OR 'azilsartan'/exp OR azilsartan	72,787
#5	#1 OR #2 OR #3 OR #4	175,663
#6	acute AND ('pancreatitis'/exp OR pancreatitis)	74,057
#7	#5 AND #6	557

Appendix 3 

Appendix 4 

Appendix 5 

Appendix 6 

Appendix 7 

Appendix 8 

Appendix 9 

Appendix 10 
